# Dr. Walker, on Mr. Ring's Case of Malformation

**Published:** 1805-05-01

**Authors:** John Walker

**Affiliations:** Salisbury Square


### *To the Editors of the Medical and Physical Journal.*

GENTLEMEN,

OF all the gratifications to the human heart, that of flattery, when not administered *ad nauseam*, is one of the most delicious, and a large dose of it will often be received without any symptom of nausea being produced. How have I seen a whole bar stretch out their necks, on the learned judge making some trifling remark! If not nauseated by their yawning ears he must have felt himself an oracle. How unceremoniously ye have brought forward John Ring's singular case of malformation of the heart; while contemporary journalists (Med. and Chirurg. Rev. July, 1803) speaking of a morbid heart, 'hope to be rather commended commended than blamed for raising the expectation of the public to receive the history of the case from the able practitioner, who alone can give it accurately!' Why did you not do your 'Constant Correspondent' the credit of mentioning, since he has been silent on it himself, that he had given his prognosis to professional men as well as to the mother? I know not whether I am likely to offend or to please him, in offering some further observations on his very curious case.

From the appearances on dissection, he says, it is not surprising that the infant did not survive the period (one year) of her life; 'it is rather a wonder that she lived so long.' It is a wonder, I grant, that she lived so long; indeed, with me, there is cause of astonishment in her living *ex utero* at all. Why did she not expire at the time of the birth, immediately on ceasing to derive sanguification (oxygenation) from the placenta? and why, having survived a few respirations, did she not go on to experience, by some spontaneous effort of nature, an accommodation of the whole system to the condition of the amphibia? I say by some spontaneous effort, because we do not recognise in this very extraordinary case of malformation, a condition of thoracic viscera that would sustain the life even of a cold blooded animal;* nor in fact, does it seem to me possible to conceive in what way the effects of the confusion of parts could be obviated by any, the most extra-

* Amphibia and fishes, different from mammalia and birds, are said to have only one ventricle and one auricle for the circulation of their comparatively cold blood. But the heart of some of the amphibia seems to be so constructed as to act as a single heart, when the animal is under water, though in the air it may be capable of effecting the long circulation.

"In a water tortoise, (says Cheselden in his Anatomy of the Human Body, book iv. near the end of chap. iii.) which I had an opportunity of examining with that most dexterous and indefatigable anatomist Dr. Douglas, I found the two ventricles of the heart but half divided by a septum, and in the beginning of the pulmonary artery, several strong muscular rings, a little distant from each other; each of which, by contracting, would be capable of resisting a part of that blood, which otherwise would have been thrown into the lungs, when they were under water; and this blood, so obstructed, must necessarily be thrown into the aorta, the two ventricles being in a manner one common cavity; and when they are out of the water, this communication of ventricles will suffer but little confusion of the blood which flows into the ventricles, because each ventricle receiving and discharging the same quantity of blood, at the same time, they will balance each other, and thereby such a mixture will be very much prevented."

extraordinary, efforts of nature. To both auricles of the heart, blood arives from the general system. The inferior cava pours its blood to the left side; there are two superior cavæ, and one of these pours its blood also to the left; the other, in the usual way, to the right. Here then are two currents of carbonated blood arriving at the left side of the heart, while only one arrives at the right, from which the pulmonary artery, very small, carries blood to the lungs, whence it makes its way to the left side. The description (page 121) shews that this last little renovated stream was liable to be mixed with the carbonated mass through all the cavities of the heart. Well might the complexion in some parts be of a lead colour; in others of a bluish purple. I am astonished that the child survived even an hour, the great change from the fœtal to the breathing state. Does not the case afford a proof that the animal is most tenacious of life in the early stages of its existence, when the important organs of brain, heart, liver, &c. are of the largest size, in proportion with other parts, particularly the parts of voluntary motion?

In the Blue Boy, in Sandifort's Collection, and in other similar cases where the subjects have even lived to approach puberty, direct communication, by an aperture, between the ventricles, has been discovered by dissection. This well explains the possibility, though not the necessity of unoxygenated blood getting into the aorta. From Cheselden's Observations on the Tortoise, I naturally enough suppose that the imperfection in the septum would not necessarily produce the morbid effect; that the septum might even be completed after birth; or, that the superior force of the left ventricle preventing this, and thus having the opportunity of acting, though the aperture, on the pulmonary system might even enlarge it, and keeping some part of the blood performing a short circulation through the lungs, hyperoxygenate the stream before its getting into the aorta, and thus produce the florid instead of the livid complexion. These considerations induce me to believe,— that the common case of malformation of the heart, in which the aorta arises from both ventricles, a deficiency in the septumof the ventricles being also found at the part where the aorta arises,— that this case of aperture, or deficiency, in the septum of the ventricles, must have arisen from a morbid state of the pulmonary vessels, a want of capacity in them to transmit the whole vital stream; and that hence unoxygenated blood has been forced through the septum, and hence must continue to be propelled through the general system by the contraction of the the left as well as of the right ventricle. The charming description of the structure of the healthy heart, and of the most curiously mechanical part of its physiology, if it may be so deemed, by Everard Home, does much increase my astonishment and surprise at the human subject's continuing to live under any, the least, irregularity in the structure of this vital organ. Your contemporaries promising themselves so much gratification 'from the present knowledge of the action of air (oxygen) in the lungs, on the thousand questions occurring to a well informed and inquisitive mind,' does not excite in me any ardent hopes of complete information. Indeed, if the blood were only sent to the lungs for the purpose of being fanned or cooled by the inspired air, or for the purpose of having a hot and noxious vapour carried off from it, all the motions of the heart would be as necessary for the preservation of the animal as they are at present. Respiration would be the same. Your's, respectfully,

*Salisbury Square, 17, iv. 1805.* JOHN WALKER.

